# Incremental Value of Cardiovascular CT in Neonates and Infants with Congenital Heart Disease: An Integrated Multimodality Imaging Approach

**DOI:** 10.3390/medsci14060687

**Published:** 2026-10-20

**Authors:** Elettra Pomiato, Alice Pozza, Irene Cattapan, Annachiara Cavaliere, Elena Reffo, Martina Avesani, Biagio Castaldi, Raffaella Motta, Valeria Pergola, Giovanni Di Salvo

**Affiliations:** 1 Paediatric Cardiology Unit, Department of Woman’s and Child’s Health, University of Padua, Via Giustiniani 2, 35128 Padova, Italy; 2 Neuroradiology Unit, Department of Woman’s and Child’s Health, University of Padua, Via Giustiniani 2, 35128 Padova, Italy; 3 Radiology Unit, Azienda Ospedale-Università Padova, Via Giustiniani 2, 35128 Padova, Italy; 4 Cardiology Unit, Cardio-Thoracic-Vascular, and Public Health Department, Padova University Hospital, Via Giustiniani 2, 35128 Padova, Italy

**Keywords:** congenital heart disease, cardiovascular computed tomography, echocardiography, neonates, infants, multimodality imaging, surgical planning

## Abstract

**Background:** Transthoracic echocardiography (TTE) is the first-line imaging modality in neonates and infants with congenital heart disease (CHD), whereas cardiovascular computed tomography (CCT) is generally reserved for selected questions that remain unresolved after echocardiography. We evaluated the incremental diagnostic contribution of CCT within this multimodality imaging pathway. **Methods:** We retrospectively reviewed consecutive patients aged <1 year with CHD who underwent CCT after multidisciplinary review because TTE was inconclusive or additional anatomic definition was required for surgical planning. The primary endpoint was the incremental anatomic information provided by CCT beyond TTE. Secondary analyses included diagnostic performance against operative findings, acquisition characteristics, radiation exposure, contrast administration, and procedural safety. **Results:** Twenty-six patients were included; 14 (53.8%) were neonates. CCT was requested most frequently to assess coronary anatomy (9/26, 38.5%), followed by the pulmonary veins (4/26, 15.4%), aortic arch (3/26, 11.5%), and pulmonary arteries/MAPCAs (4/26, 15.4%). CCT provided additional anatomic characterization in 14/26 patients (53.8%), predominantly involving extracardiac vascular structures, and was discordant with TTE regarding the unresolved target anatomy in 12/26 (46.2%). Against the operative findings, TTE accuracy was 96.2% for intracardiac abnormalities, 41.2% for extracardiac abnormalities, and 11.1% for coronary abnormalities; corresponding CCT accuracy was 100%, 94.1%, and 100%. CCT directly informed the surgical approach in two patients with total anomalous pulmonary venous drainage. All examinations provided sufficient image quality to address the specific clinical question for which CCT was performed, and no contrast-related adverse events occurred. The median estimated effective dose was 3.62 mSv (IQR, 1.94–6.06). **Conclusions**: In a highly selected cohort of neonates and infants with CHD in whom expert TTE leaves a clinically relevant question unresolved, CCT may provide incremental information, particularly regarding coronary and extracardiac vascular anatomy. These findings support a targeted multimodality strategy in which CCT complements echocardiography and is tailored to the anatomic question relevant to surgical planning.

## 1. Introduction

Transthoracic echocardiography (TTE) is the cornerstone of cardiovascular imaging in neonates and infants with congenital heart disease (CHD). High-frequency transducers, excellent temporal resolution, portability, and the ability to obtain real-time hemodynamic information allow for detailed characterization of intracardiac morphology and function in most patients [1,2]. In experienced pediatric congenital programs, TTE therefore provides sufficient information for diagnosis and surgical decision-making in the large majority of cases.

However, no single imaging technique resolves every anatomic question in complex CHD. Cardiovascular computed tomography (CCT) offers high spatial resolution, rapid acquisition, three-dimensional assessment of vascular relationships, and robust visualization of structures that may be difficult to define with ultrasound, including the coronary arteries, pulmonary veins, branch pulmonary arteries, major aortopulmonary collateral arteries (MAPCAs), and complex aortic arch anatomy [3,4,5,6]. Contemporary multimodality imaging is therefore best viewed as a complementary strategy in which each technique is used to answer the questions it is best suited to address [7,8].

This concept is particularly relevant in neonates and infants, in whom the threshold for additional imaging must balance the potential clinical value of CCT against the risks of ionizing radiation, iodinated contrast administration, and the possible sedation. Modern wide-detector scanners, prospective electrocardiographic triggering, individualized tube voltage, and iterative reconstruction have substantially improved image quality and dose efficiency in pediatric CHD [6,9,10]. Accordingly, the clinically relevant question is not whether CCT is globally more accurate than echocardiography, but rather when CCT adds actionable anatomic information after a high-quality TTE examination.

The aim of this study was therefore to assess the incremental diagnostic value of CCT within an integrated preoperative imaging pathway for neonates and infants with CHD who were referred for CCT after multidisciplinary review because TTE was considered inconclusive or because further anatomic definition was required. We additionally evaluated the diagnostic performance of both modalities against operative findings and described contemporary CCT acquisition, radiation exposure, contrast administration, and procedural safety.

## 2. Materials and Methods

This retrospective single-center study included neonates and infants with CHD evaluated at a tertiary pediatric cardiac center between 2019 and 2025. Patients were discussed by the institutional congenital heart disease multidisciplinary team (MDT) for preoperative planning. The center performs approximately 300 pediatric cardiac surgical procedures annually.

As part of the institutional diagnostic pathway, all patients underwent comprehensive TTE before MDT discussion. CCT was not performed routinely. It was requested as a second-line examination when the MDT considered that TTE did not fully resolve a clinically relevant anatomic question or when additional anatomic detail was required for operative planning. This referral strategy was deliberately selective and represents the real-world use of CCT as an adjunct to echocardiography rather than as a competing first-line test. Inclusion criteria were: (1) diagnosis of CHD; (2) age < 1 year at the time of both TTE and CCT; (3) CCT performed after MDT review because of an unresolved or incompletely characterized anatomic question; and (4) subsequent cardiac surgery allowing for a comparison with intraoperative anatomy. Consecutive patients fulfilling these criteria were included. Demographic and clinical characteristics, CHD diagnosis, indication for CCT, previous cardiac surgery or intervention, TTE findings, CCT findings, scan parameters, radiation metrics, contrast administration, venous access, sedation, and adverse events including contrast-related adverse reactions and post-contrast kidney injury [11] were retrospectively collected. TTE and CCT data were retrieved from the original echocardiography and CCT reports and images. Surgical findings were obtained from the clinical record. Because of the retrospective nature of the study, the operative team was not blinded to the imaging findings. Any discrepancies between imaging and operative findings were identified by comparing imaging documentation with the operative reports.

**Figure 1:**
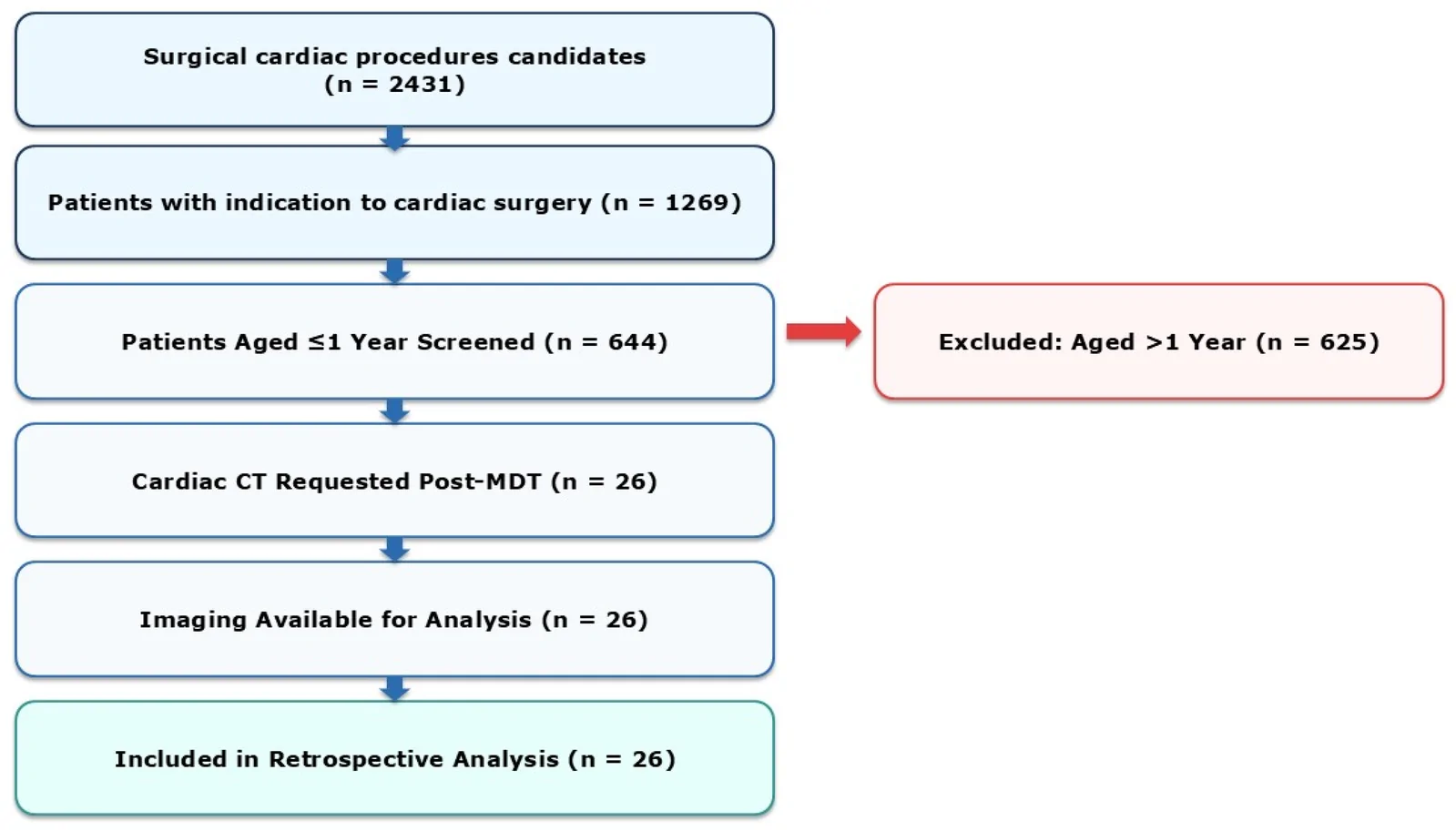
STROBE flow diagram showing patient selection, exclusions, and inclusion in the study.

The primary outcome was the incremental anatomic information provided by CCT beyond TTE. This was defined using four hierarchical levels, and each patient was assigned to the highest applicable category: (A) confirmation; (B) additional anatomic detail without change in diagnosis or management (minor incremental information); (C) revision of the TTE diagnosis of the target anatomy, with no change in surgical approach (major incremental information with no change in operative management); and (D) diagnostic revision resulting in a change in surgical approach (major incremental information with management impact). The secondary outcome was the diagnostic performance of TTE and CCT, assessed by sensitivity, specificity, and accuracy using invasive findings as the reference standard. Diagnostic pathways for neonates and infants are summarized in Figure 1. This retrospective cohort study was reported in accordance with the Strengthening the Reporting of Observational Studies in Epidemiology (STROBE) guidelines.

### 2.1. Transthoracic Echocardiography

Comprehensive two-dimensional TTE was performed by pediatric cardiologists experienced in CHD using a Vivid E9 ultrasound system (GE Healthcare, Horten, Norway) with S12, S6, or SV6c transducers selected according to patient size and acoustic conditions, in accordance with contemporary pediatric and congenital echocardiographic recommendations [1,12]. Images were interpreted at the time of examination, and when required, reviewed offline using EchoPAC (version 112.99, GE Healthcare, Horten, Norway).

### 2.2. Cardiovascular CT

CCT examinations were performed on a 320-detector-row scanner (Aquilion ONE, Canon Medical Systems, Otawara, Japan). Acquisition parameters were individualized to the diagnostic question and patient characteristics, with electrocardiographic triggering or gating used as clinically appropriate. Examinations were jointly interpreted by a CCT-accredited cardiologist and a cardiovascular radiologist. Tube voltage, acquisition mode, dose-length product (DLP), estimated effective dose, iodinated contrast agent and volume, venous access, sedation, and procedural complications were recorded. Radiation exposure in mSv was calculated using published k-factors for chest exposure in newborn and children up to 1 year of age [13].

### 2.3. Statistical Analysis

Categorical variables are reported as counts and percentages and continuous variables as mean ± standard deviation or as median and interquartile range (IQR), according to distribution. Diagnostic performance was analyzed on a patient-level basis for the assessment of intracardiac, extracardiac, and coronary artery anatomy. Sensitivity, specificity, and accuracy of TTE and CCT were calculated using intraoperative findings as the reference standard. The 95% confidence intervals were calculated using the exact Clopper–Pearson method. Agreement between TTE and CCT was assessed with Cohen’s kappa. Cohen’s κ was reported with 95% confidence intervals calculated using the Fisher z transformation. Two-sided *p*-values for testing κ = 0 were calculated using an asymptotic z test. Because the cohort was small and intentionally enriched for cases with inconclusive or incomplete echocardiographic assessment, diagnostic-performance estimates were considered descriptive and were not used to infer overall superiority of one modality over the other. A two-sided *p* value < 0.05 was considered statistically significant for exploratory analyses. Analyses were performed using jamovi version 2.6.26.

## 3. Results

During the study period, approximately 2400 patients underwent cardiac surgery at our center. Twenty-six consecutive neonates and infants (1.1% of the surgical population) fulfilled the study criteria and underwent CCT after MDT review because an anatomic question remained unresolved or incompletely characterized by TTE. Fourteen patients (53.8%) were neonates and 10 (38.5%) were male. Baseline diagnoses and CCT indications are summarized in Table 1; CCT-specific data, including the acquisition protocol and contrast administration, are presented in Table 2; patient-level details are available in the Supplementary Materials.

CCT was feasible and answered the clinical question in all 26 patients, including 3 patients (11.5%) receiving extracorporeal membrane oxygenation at the time of scanning. No examination was terminated because of instability or technical failure, and no immediate procedural complication was recorded.

The most frequent indication for CCT was assessment of coronary artery anatomy in 9/26 patients (34.6%). Other indications included pulmonary venous anatomy in 4/26 (15.4%), aortic arch anatomy in 3/26 (11.5%), pulmonary artery branches in 2/26 (7.7%), MAPCAs in 2/26 (7.6%), and other complex questions in 5/26 (19.2%). Heart rate exceeded 100 beats/min in all patients. Most examinations (22/26, 84.6%) were performed at 80 kVp and 2 (7.7%) at 100 kVp; tube-voltage information was unavailable for the remaining examinations. Prospective acquisition was used in 11/26 (42.3%) and retrospective acquisition in 8/26 (30.8%); acquisition mode was not retrievable in 7/26 (26.9%). Retrospective gating was selected when broader phase coverage or more challenging anatomic definition was required, including the evaluation of MAPCAs, heterotaxy, coronary arteries, and total anomalous pulmonary venous drainage (TAPVD).

Median DLP was 92.8 (IQR 49.6–155) mGy·cm and median estimated effective dose was 3.62 (IQR 1.94–6.06) mSv. As expected, radiation exposure was significantly higher with retrospective than with prospective gating (10.5 [IQR 9.35–12.0] vs. 2.92 [IQR 2.45–3.52], *p* = 0.002). Iodinated contrast was administered in all patients, most commonly through a peripheral venous cannula (19/26, 73.1%); central venous and umbilical venous access were used in 5/26 (19.2%) and 2/26 (7.7%), respectively. Iohexol was used in 25/26 examinations (96.2%) and iomeprol in 1/26. Median contrast volume was approximately 1.92 mL/kg (IQR 1.68–2.58). All 26 patients received sedation, and 4 were also intubated. No cases of contrast extravasation, immediate contrast-related adverse reaction, or post-contrast kidney injury were observed. CCT acquisition characteristics are summarized in Table 3.

### 3.1. Incremental Diagnostic Contribution of CCT

CCT confirmed the TTE findings (group A) in 12 patients (46.2%) while providing only minor additional anatomic details (group B) involving branch pulmonary artery and coronary anatomy in 2 patients (7.7%). CCT provided major additional anatomic information in 12 patients (46.2%). In 10 patients (38.5%), this additional information did not alter the surgical plan (Group C), whereas in 2 (7.7%), it substantially influenced surgical management (Group D). Overall, the incremental information concerned extracardiac vascular anatomy in 12 patients (Groups C and D, 46.2% of the total cohort), emphasizing the complementary role of CCT for structures that are less consistently visualized by TTE.

**Figure 2:**
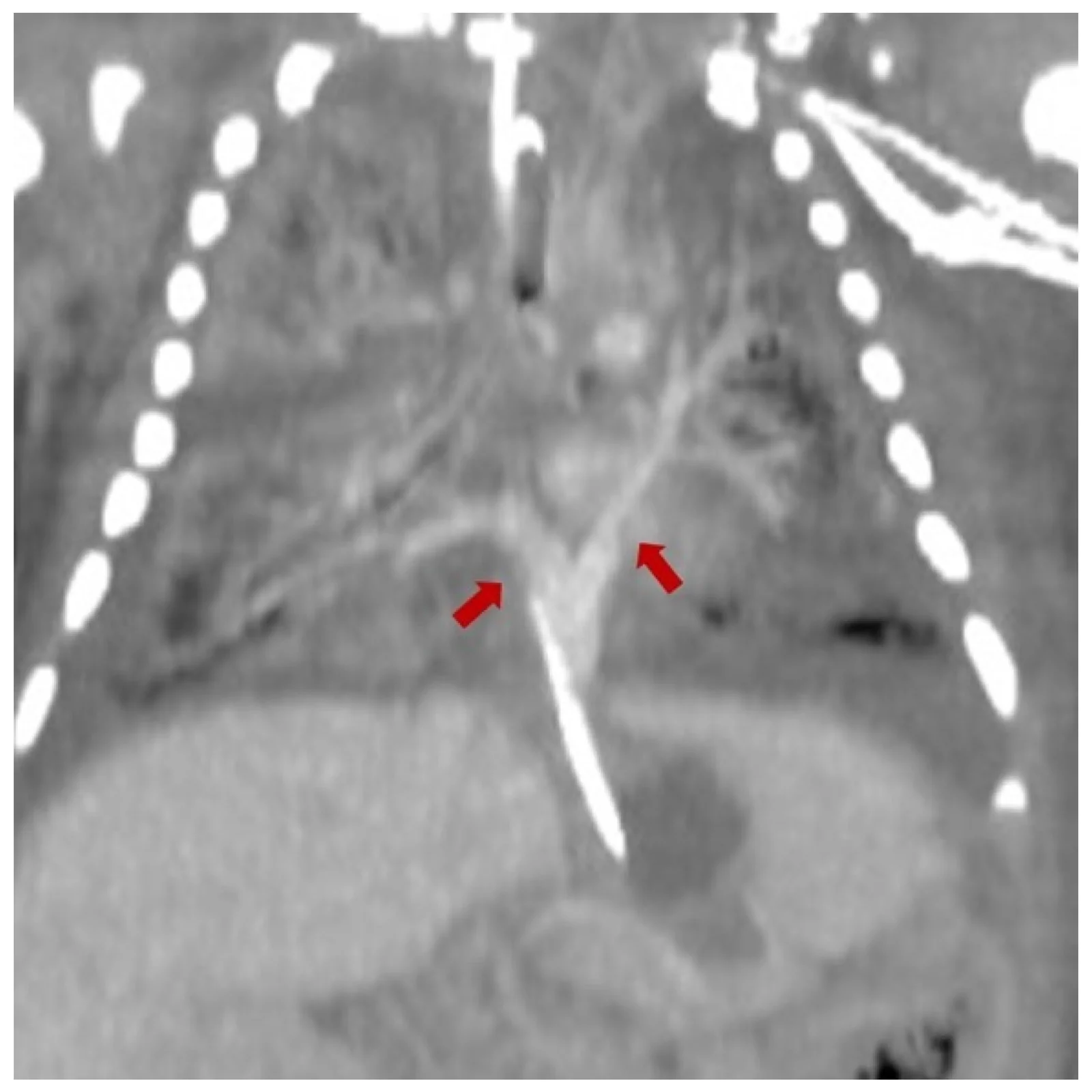
CCT showing infracardiac TAPVD (arrows).

**Figure 3:**
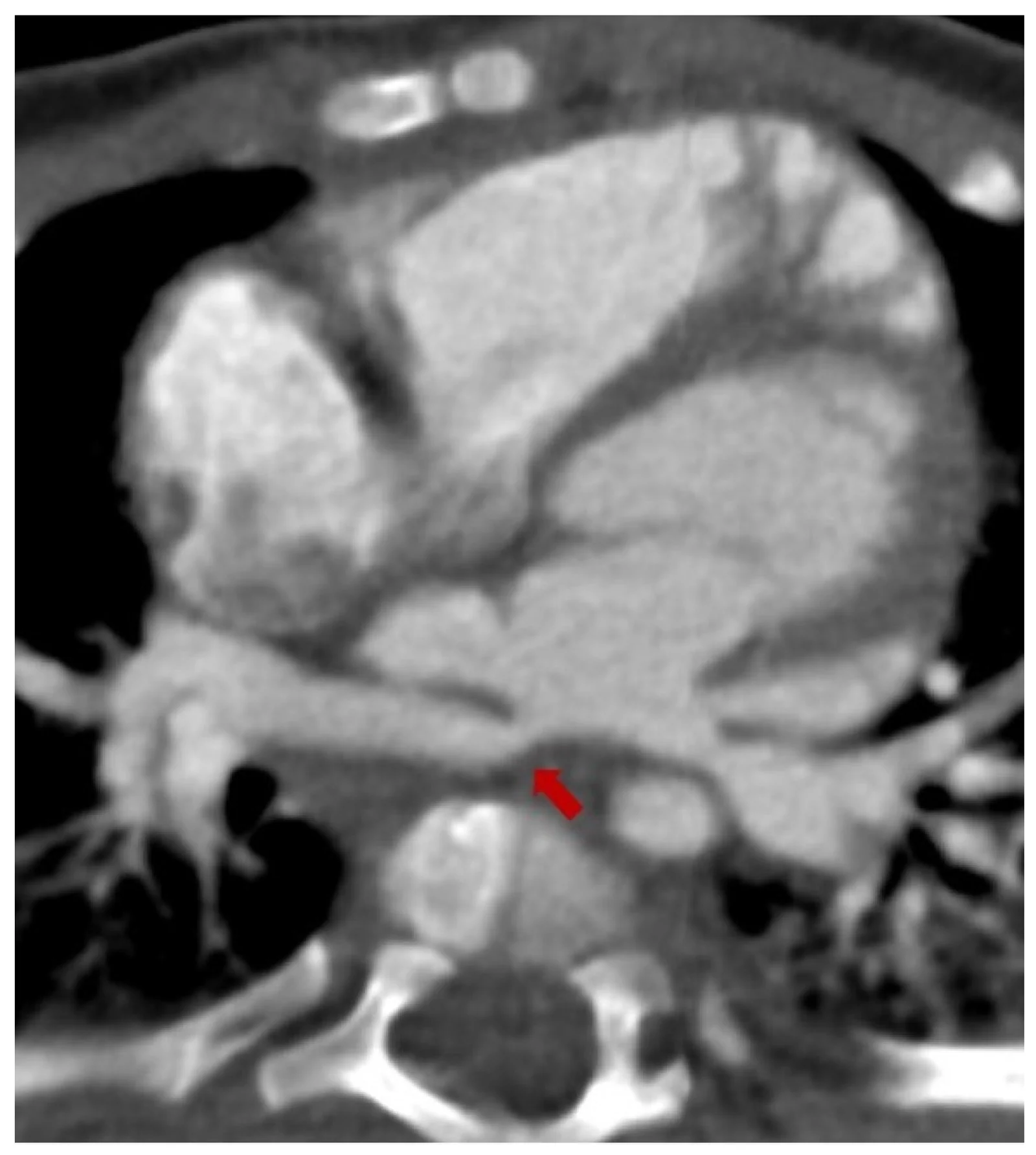
CCT showing collector stenosis following TAPVD repair (arrow).

CCT had a direct impact on operative planning in 2/26 patients (7.7%), both of whom had TAPVD. In both cases, cross-sectional definition of pulmonary venous drainage was pivotal in selecting the surgical approach. The first was a neonate without a prenatal diagnosis who was transferred to our center on veno-arterial ECMO for unexplained right ventricular disfunction and pulmonary hypertension. TTE subsequently raised suspicion of anomalous pulmonary venous drainage. CCT confirmed the diagnosis of obstructive infracardiac TAPVD (Figure 2), leading to cardiac surgery. The second patient had a history of surgically corrected infracardiac TAPVD. Two months after surgery, the patient experienced worsening respiratory symptoms, and a poor acoustic window limited echocardiographic assessment. CCT demonstrated stenosis of the pulmonary venous collector (Figure 3), which was subsequently treated.

### 3.2. Diagnostic Performance of TTE and CCT

In total, 26 patients underwent an assessment of intracardiac anatomy, 17 patients underwent an assessment of extracardiac anatomy, and 9 patients underwent an assessment of coronary artery anatomy. Using intraoperative findings as the reference standard, TTE sensitivity, specificity, and accuracy were 95.7% (95% CI, 78.1–99.9), 100% (95% CI, 29.2–100), and 96.2% (95% CI, 80.4–99.9), respectively, for intracardiac abnormalities; 35.7% (95% CI, 12.8–64.9), 66.7% (95% CI, 9.4–99.2), and 41.2% (95% CI, 18.4–67.1) for extracardiac abnormalities; and 33.3% (95% CI, 0.8–90.6), 0% (95% CI, 0.0–45.9), and 11.1% (95% CI, 0.3–48.2) for coronary abnormalities. The corresponding values for CCT were 100% (95% CI, 85.2–100), 100% (95% CI, 29.2–100), and 100% (95% CI, 86.8–100) for intracardiac anatomy; 92.9% (95% CI, 66.1–99.8), 100% (95% CI, 29.2–100), and 94.1% (95% CI, 71.3–99.9) for extracardiac thoracic anatomy; and 100% (95% CI, 29.2–100), 100% (95% CI, 54.1–100), and 100% (95% CI, 66.4–100) for coronary anatomy. Agreement between TTE and CCT was high for intracardiac anatomy (96%; κ = 0.835, 95% CI, 0.171–0.978; *p* < 0.001), but was substantially lower and not statistically significant for extracardiac anatomy (47%; κ = 0.084, 95% CI, −0.232–0.384; *p* = 0.622) and coronary anatomy (11%; κ = −0.500, 95% CI, −0.881–0.276; *p* = 0.023). These estimates should be interpreted in the context of the deliberately selected population referred for CCT after an incomplete or inconclusive echocardiographic assessment. The coronary-category κ should be interpreted cautiously given the limited sample size, which may result in substantial uncertainty around the estimated agreement. Contingency Tables are available in the Supplementary Materials.

## 4. Discussion

The principal finding of this retrospective exploratory study is that within a deliberately selective multimodality pathway, CCT added clinically relevant anatomic information in more than half of the neonates and infants referred after expert TTE left a question unresolved or incompletely characterized. The added value was concentrated in coronary and extracardiac vascular anatomy, whereas TTE remained highly reliable for intracardiac morphology. These findings support a complementary model of imaging: echocardiography provides the first-line structural and hemodynamic assessment, and CCT is deployed selectively to resolve specific anatomic uncertainties with potential relevance to surgery.

This distinction is important because a head-to-head comparison of TTE and CCT would not reflect how the two modalities are used clinically in early-life CHD. TTE is non-ionizing, portable, repeatable, and provides real-time functional and hemodynamic information; for these reasons, it remains the foundational modality in neonates and infants [1,2]. In our center, only a small minority of surgical patients required CCT after MDT review, underscoring that cross-sectional imaging was used as a targeted problem-solving tool rather than as routine duplication of echocardiography.

The pattern of incremental findings is also clinically plausible. Coronary origin and course, pulmonary venous connections, branch pulmonary arteries, MAPCAs, and complex aortic arch relationships may be difficult to fully characterized by TTE because of vessel size, orientation, overlying air-filled lung, postoperative anatomy, or limited acoustic windows. CCT is particularly well-suited to these structures because of its isotropic spatial resolution, rapid volumetric acquisition, and capacity for multiplanar and three-dimensional reconstruction [3,4,5,6].

Our referral profile is consistent with previous pediatric CCT series. Saengsin et al. reported that pulmonary artery definition, aortic anatomy, pulmonary veins, and coronary arteries were common indications for CCT in infants and demonstrated a meaningful impact on management [5]. Studies comparing TTE with CT angiography across broader CHD populations have likewise shown the greatest incremental yield of CT for extracardiac vascular abnormalities [14,15]. Our study extends this concept to a highly selected cohort restricted to the first year of life and anchored to an MDT-based preoperative workflow.

The diagnostic-performance analysis should therefore be interpreted as supportive in selected cases rather than as evidence that CCT is globally superior to echocardiography. The high TTE accuracy for intracardiac anatomy confirms the strength of echocardiography in this age group. Conversely, lower TTE accuracy and agreement for extracardiac and coronary anatomy reflect both the intrinsic limitations of ultrasound for these targets, and importantly, referral enrichment: patients were selected for CCT precisely because these questions had not been fully resolved. This perspective and verification bias make direct modality comparisons intrinsically asymmetric. The clinically relevant observation is that targeted CCT resolved uncertainty in 53.8% of the cohort, but directly influenced operative management in two cases of TAPVD.

Coronary anatomy represented the most frequent indication for CCT. Contemporary ECG-synchronized CT can delineate coronary origins and proximal courses in infants despite high heart rates [16,17]. In D-transposition of the great arteries, CT may provide additional preoperative information regarding coronary patterns and spatial relationships when TTE is uncertain [18]. In our series, TTE generally raised the suspicion of abnormal coronary anatomy, whereas CCT was used to define the anatomy more confidently before surgery. This is a prototypical example of complementary rather than competitive imaging.

The feasibility of CCT in this cohort is noteworthy. All examinations were diagnostic despite heart rates >100 beats/min, and CCT was successfully performed even in clinically fragile patients, including three receiving extracorporeal membrane oxygenation. Wide-detector technology is particularly advantageous in small children because it enables rapid coverage with reduced motion sensitivity and can improve the visualization of small-caliber cardiovascular structures [6,9]. Nevertheless, acquisition should remain tailored to the specific diagnostic target, patient size, heart rate, and need for temporal information.

Radiation exposure remains a central consideration in neonates and infants because of their greater lifetime attributable risk. Current consensus emphasize justification of every examination and optimization according to the ALARA principle, including individualized scan length, tube voltage, ECG-synchronization strategy, and iterative reconstruction [6,10]. The median estimated effective dose in our cohort was 3.62 mSv, which is higher than the reference value of 1 mSV reported in consensus recommendations [6]. This value reflects a heterogeneous, clinically complex population in whom some examinations required retrospective acquisition. It should not be interpreted as a target dose; rather, it highlights the need to tailor the acquisition strategy to the clinical question, emphasizing appropriate referral for CCT to minimize unnecessary radiation exposure while continuing to implement dose optimization strategies whenever possible.

All patients received sedation. Although modern rapid-acquisition techniques can reduce or avoid sedation in many neonates, motion control remains important in selected infants, particularly when detailed coronary or complex vascular assessment is required [6,8,19]. Decisions regarding sedation should therefore be individualized and integrated into the overall procedural risk–benefit assessment.

Iodinated contrast was administered in all patients, most often through peripheral venous access, without any observed extravasation, immediate contrast reaction, or post-contrast kidney injury. Severe iodinated contrast reactions are uncommon in children [20], and contemporary observational data suggest that the risk of acute kidney injury after intravenous contrast is strongly influenced by baseline renal and clinical risk factors rather than contrast exposure alone [21]. Nevertheless, the smallest contrast volume compatible with diagnostic enhancement should be used, with careful attention to renal function, hydration, venous access, and concurrent nephrotoxic therapies.

## 5. Limitations

This study has several limitations. First, its retrospective, single-center design and small, anatomically heterogeneous sample limit statistical precision and generalizability, particularly to centers with different referral thresholds or levels of congenital echocardiographic and/or CT expertise. Second, the cohort was deliberately restricted to patients in whom TTE left an unresolved anatomic question and who subsequently underwent surgery. Thus, diagnostic-performance estimates are based on small denominators and a highly selected cohort, making them susceptible to referral, spectrum, and verification bias. In particular, the coronary-category κ should be interpreted cautiously given the limited sample size, which may result in substantial uncertainty around the estimated agreement. They should therefore not be interpreted as evidence of general superiority of CCT over TTE or extrapolated to unselected populations. Third, although the primary endpoint was classified according to predefined hierarchical criteria, its retrospective assessment introduces the potential for subjectivity. Fourth, operative findings served as the reference standard; however, surgeons were aware of the CCT findings, introducing potential review and selection bias. Fifth, acquisition parameters were unavailable for a proportion of examinations, limiting the representativeness of the reported technical and dosimetric data. In addition, effective dose was estimated using a DLP-based method, which is imprecise in infants. Finally, patients who underwent CCT but did not proceed to surgery, as well as those evaluated with cardiac magnetic resonance, were excluded; therefore, this study does not address the comparative role of alternative second-line imaging modalities.

## 6. Conclusions

In neonates and infants with CHD, TTE remains the first-line imaging modality and is sufficient for preoperative assessment in the vast majority of patients. When a clinically relevant anatomic question persists after expert echocardiography, targeted CCT can provide substantial incremental value, particularly for coronary arteries and extracardiac vascular structures. In this selected cohort, CCT added anatomic information in 53.8% of patients and directly informed surgical planning in selected cases, while all examinations were diagnostic and no contrast-related adverse events were observed. A question-driven, MDT-based multimodality strategy can therefore leverage the complementary strengths of TTE and CCT while minimizing unnecessary radiation and contrast exposure.

## Figures and Tables

**Table 1:** Baseline characteristics and indications for cardiovascular CT in 26 patients with congenital heart disease.

		N (%)
Age	<1 month	14 (53.8)
	1 month–1 year	12 (46.2)
Sex (male)		10 (38.5)
Baseline CHD	TOF	6 (23.1)
	Arch and CoA	4 (15.4)
	D-TGA	3 (11.5)
	DORV	3 (11.5)
	TAPVD	4 (15.4)
	HLHS	3 (11.5)
	Heterotaxy/crisscross heart	3 (11.5)
Previous cardiac surgery		4 (15.4)
Indication for CCT	Coronary arteries	9 (34.6)
	Pulmonary veins	4 (15.4)
	Aortic arch anatomy	3 (11.5)
	PA branches	2 (7.7)
	MAPCAs evaluation	2 (7.7)
	Other reasons	6 (23.1)
CCT Request	CHD Cardiac Surgeon	13 (50%)
	CHD Cardiologist	13 (50%)

CHD—congenital heart disease, TOF—tetralogy of Fallot, Arch—abnormalities including interrupted aortic arch, CoA—aortic coarctation, D-TGA—transposition of the great arteries with dextroposition, DORV—double outlet right ventricle, TAPVD—total anomalous pulmonary venous drainage, HLHS—hypoplastic left heart syndrome, PA—pulmonary artery, MAPCAs—major aortopulmonary collaterals, CCT—cardiovascular CT.

**Table 2:** Cardiovascular CT acquisition characteristics, radiation exposure, and contrast administration.

		N (%)/Median (IQR)
Age (days)		20.0 (10–116)
Weight (kg)		3.54 (3.01–5.75)
HR (bpm)		138 (123–148)
DLP (mGy·cm)		92.8 (49.6–155)
Radiation dose (mSv)		3.62 (1.94–6.06)
Image acquisition	Prospective	11 (42.3)
	Retrospective	8 (30.8)
	Not available	7 (26.9)
ECMO		3 (11.5)
CCT Contrast Agent	Iohexol	25 (96.2)
	Iomeprol	1 (3.8)
Venous access	Peripheral	19 (73.1)
	CVC	5 (19.2)
	UVC	2 (7.7)
CA dose (mL/kg)		1.92 (1.68–2.58)

DLP—dose-length product (mGy·cm), CA—iodine contrast agent, CVC—central venous catheter, UVC—umbilical venous access.

**Table 3:** Incremental diagnostic contribution of CCT in 26 neonates and infants with CHD.

Category	Definition	N (%)
A. Confirmation	CCT confirmed the TTE diagnosis of the target anatomy without additional clinically relevant detail	12 (46.2)
B. Additional detail	TTE diagnosis confirmed; CCT added anatomic detail relevant to planning without changing diagnosis or management	2 (7.7)
C. Diagnostic revision	CCT corrected or substantially refined the TTE diagnosis of the target anatomy without changing the surgical approach	10 (38.5)
D. Management change	Diagnostic revision that altered the planned surgical approach	2 (7.7)

CCT—cardiovascular CT, TTE—transthoracic echocardiogram.

## Data Availability

The original contributions presented in this study are included in the article/Supplementary Materials. Further inquiries can be directed to the corresponding author.

## Supplementary Materials

The following supporting information can be downloaded at: https://www.mdpi.com/article/10.3390/medsci14060687/s1, Table S1: Patient-level diagnoses, prior interventions, and indications for cardiovascular CT; Table S2: Contingency Table for TTE intracardiac findings against intraoperative findings; Table S3: Contingency Table for TTE extracardiac findings against intraoperative findings; Table S4: Contingency Table for TTE coronary artery anatomy against intraoperative findings; Table S5: Contingency Table for CCT intracardiac anatomy against intraoperative findings; Table S6: Contingency Table for CCT extracardiac findings against intraoperative findings; Table S7: Contingency Table for CCT coronary artery anatomy against intraoperative findings.

## Abbreviations

The following abbreviations are used in this manuscript:

TTE	transthoracic echocardiography
CHD	congenital heart disease
CCT	cardiovascular computed tomography
MAPCAs	major aortopulmonary collateral arteries
MDT	multidisciplinary team
DLP	dose-length product
TAPVD	total anomalous pulmonary venous drainage
TOF	tetralogy of Fallot
CoA	aortic coarctation
D-TGA	transposition of the great arteries with dextroposition
DORV	double outlet right ventricle
HLHS	hypoplastic left heart syndrome
PA	pulmonary artery
IAA	interrupted aortic arch
AVSD	atrio-ventricular septal defect
CA	iodine contrast agent
CVC	central venous catheter
UVC	umbilical venous access
