# Supplementary material for: Incremental Value of Cardiovascular CT in Neonates and Infants with Congenital Heart Disease: An Integrated Multimodality Imaging Approach
Source: Med Sci (Basel). 2026 Oct 20;14(6):687. doi: 10.3390/medsci14060687 (PMC13707324; doi:10.3390/medsci14060687)
Supplement: Supplementary file 1 [file medsci-14-00687-s001.zip › medsci-4548995-supplementary.pdf]

Supplementary materials

Supplementary Table S1. Patient-level diagnoses, prior interventions, and indications for cardiovascular CT

ID	Age Group	Diagnosis	Previous cardiac surgery/intervention	CCT indication
#1	Infant	TOF	NO	Coronary arteries
#2	Newborn	IAA with VSD	NO	Arch anatomy
#3	Newborn	TOF PA type	NO	MAPCAs
#4	Infant	TAPVD	NO	PV drainage
#5	Newborn	D-TGA with VSD and PS	NO	Coronary arteries
#6	Infant	Heterotaxy sdr	NO	Venous drainage
#7	Newborn	IAA	YES	Arch anatomy
#8	Newborn	TOF PA type	NO	MAPCAs
#9	Infant	DORV-TGA	NO	Coronary arteries
#10	Newborn	TOF PA type	NO	Coronary arteries
#11	Infant	TOF	NO	Coronary arteries
#12	Newborn	HLHS	YES	PA branches
#13	Infant	Heterotaxy sdr	NO	Coronary arteries
#14	Newborn	IAA	NO	Arch anatomy
#15	Infant	DORV-AVSD	NO	Intracardiac anatomy
#16	Newborn	D-TGA	NO	Coronary arteries
#17	Infant	HLHS	YES	PA branches
#18	Infant	CoA with VSD	YES	VSD anatomy
#19	Infant	TAPVD	YES	PV drainage
#20	Infant	DORV-TGA	YES	Post CS
#21	Newborn	D-TGA	NO	Coronary arteries
#22	Newborn	TAPVD	NO	PV drainage
#23	Infant	Suspected coronary anomaly	NO	Coronary arteries
#24	Newborn	TOF PA type, double aortic arch	NO	Pulmonary vasculature
#25	Newborn	criss cross heart	NO	Outflow tracts
#26	Newborn	TAPVD	NO	PV drainage

TOF tetralogy of Fallot, IAA interrupted aortic arch, CoA aortic coarctation, D-TGA transposition of the great arteries with dextroposition, DORV double outlet right ventricle, PA type pulmonary atresia type, MAPCAs major aortopulmonary collaterals, TAPVD total anomalous pulmonary venous drainage, AVSD atrio-ventricular septal defect, HLHS hypoplastic left heart syndrome, PA pulmonary artery, CS cardiac surgery, , sdr syndrome

Supplementary Table S2 Contingency Table for TTE intracardiac findings against intraoperative findings

	Operative findings		
TTE Findings	1	0	Total
1	22	0	22
0	1	3	4
Total	23	3	26

TTE transthoracic echocardiogram

Supplementary Table S3 Contingency Table for TTE extracardiac findings against intraoperative findings

	Operative findings		
TTE Findings	1	0	Total
1	5	1	6
0	9	2	11
Total	14	3	17

TTE transthoracic echocardiogram

Supplementary Table S4 Contingency Table for TTE coronary artery anatomy against intraoperative findings

TTE Findings	Operative findings		Total
	1	0	
1	1	6	7
0	2	0	2
Total	3	6	9

TTE transthoracic echocardiogram

Supplementary Table S5 Contingency Table for CCT intracardiac anatomy against intraoperative findings

CCT Findings	Operative findings		Total
	1	0	
1	23	0	23
0	0	3	3
Total	23	3	26

CCT cardiovascular CT

Supplementary Table S6 Contingency Table for CCT extracardiac findings against intraoperative findings

	Operative findings		
CCT Findings	1	0	Total
1	13	0	13
0	1	3	4
Total	14	3	17

CCT cardiovascular CT

Supplementary Table S7 Contingency Table for CCT coronary artery anatomy against intraoperative findings

	Operative findings		
CCT Findings	1	0	Total
1	3	0	3
0	0	6	6
Total	3	6	9

CCT cardiovascular CT
